# Perspectives on cancer screening participation in a highly urbanized region: a Q-methodology study in The Hague, the Netherlands

**DOI:** 10.1186/s12889-022-14312-4

**Published:** 2022-10-15

**Authors:** Thomas H. G. Bongaerts, Frederike L. Büchner, Matty R. Crone, Job van Exel, Onno R. Guicherit, Mattijs E. Numans, Vera Nierkens

**Affiliations:** 1 Health Campus The Hague, Leiden University Medical Center, The Hague, The Netherlands; 2 Department of Public Health and Primary Care, Leiden University Medical Center, Leiden, The Netherlands; 3 Erasmus School of Health Policy & Management, Erasmus University Rotterdam, Rotterdam, The Netherlands; 4 Erasmus Centre for Health Economics Research, Erasmus University Rotterdam, Rotterdam, The Netherlands; 5 University Cancer Center Leiden - The Hague, at Haaglanden Medical Center, The Hague, The Netherlands

**Keywords:** Cancer screening, Participation, Uptake, Perspectives, Q-methodology, I-Change model

## Abstract

**Background:**

The Netherlands hosts, as many other European countries, three population-based cancer screening programmes (CSPs). The overall uptake among these CSPs is high, but has decreased over recent years. Especially in highly urbanized regions the uptake rates tend to fall below the minimal effective rate of 70% set by the World Health Organization. Understanding the reasons underlying the decision of citizens to partake in a CPS are essential in order to optimize the current screening participation rates. The aim of this study was to explore the various perspectives concerning cancer screening among inhabitants of The Hague, a highly urbanized region of the Netherlands.

**Methods:**

A Q-methodology study was conducted to provide insight in the prevailing perspectives on partaking in CSPs. All respondents were inhabitants of the city of The Hague, the Netherlands. In an online application they ranked a set of 31 statements, based on the current available literature and clustered by the Integrated Change model, into a 9-column forced ranking grid according to level of agreement, followed by a short survey. Respondents were asked to participate in a subsequent interview to explain their ranking. By-person factor analysis was used to identify distinct perspectives, which were interpreted using data from the rankings and interviews.

**Results:**

Three distinct perspectives were identified: 1). *“*Positive about participation*”*, 2). *“*Thoughtful about participation*”*, and 3). *“*Fear drives participation*”*. These perspectives provide insight into how potential respondents, living in an urbanized region in the Netherlands, decide upon partaking in CSPs.

**Conclusions:**

Since CSPs will only be effective when participation rates are sufficiently high, it is essential to have insight into the different perspectives among potential respondents concerning partaking in a CSP. This study adds new insights concerning these perspectives and suggests several ideas for future optimization of the CSPs.

## Background

The Netherlands, as many other European countries, invests considerable time and effort in hosting three population-based cancer screening programmes (CSPs) [1]. These programmes focus on cervical, breast and colorectal cancer. CSPs aim to detect cancer in an early or precursor stage and thereby improving survival via early intervention. On average, this approach is assumed to lead to a better prognosis, as well as to fewer and less severe side effects of treatment [2–5]. In the Netherlands, the screening tests of the CSPs are offered free of charge by the government to all citizens of a specific age and gender. The cervical CSP includes women aged between 30–60 and uses a Papanicolaou-smear test, a bilateral mammography is used to screen women between 50–75 years of age on breast cancer. The colorectal CSP is aimed at both women and men aged between 55–75 years, and screening is performed by a faecal immunochemical test. The National Institute for Public Health and the Environment (RIVM) and five regional screening organisations are charged with organizing and coordinating these programmes [6]. Attendance is voluntary and monitored yearly by RIVM [7–9]. Although the three CSPs show many similarities, each CSP has its unique procedures and organization, mainly due to the differences in screening methods [6].

High participation rates are essential for screening programmes to be (cost-)effective [10, 11]. According to the World Health Organization (WHO), at least 70% of the target population should be screened in order to be beneficial on population level [12–14]. Throughout Europe participation in CSPs varies substantially, yet the Netherlands is/was always known for its high screening attendance and adherence [1]. Latest published CSP attendance rates in the Netherlands, before the Covid-19 pandemic (concerning the year 2019), showed rates of 56.0%, 76.0% and 71.8% for the CSPs focused at cervical, breast and colorectal cancer, respectively [7–9]. Although the attendance rates of two programmes are above the recommended rate from WHO, there is an alarming downward trend and wide regional variation in screening uptake. In 2010, the uptake rates of the CSPs for cervical and breast cancer were 65.5% and 80.7% [7, 8]. Since the colorectal CSP has only been fully operational since 2019, it is too early to draw any conclusions on trends regarding this screening programme. At the regional level, the four largest cities of the Netherlands are among the regions with the lowest attendance rates, below the minimal effective rate of 70% for all three screening programmes [15].

In order to improve the attendance rates, it is essential to understand the motivations of citizens to participate in CSPs. A systematic review showed that earlier studies into cancer screening participation have not provided in-depth information on the underlying beliefs and motivations regarding willingness to participate in cancer screening [16]. Later studies were conducted to reveal the decision processes regarding screening participation [17, 18], but detailed understanding of the perspectives of potential participants remains limited. Furthermore, the underlying beliefs and motives to participate in CSPs could differ between subgroups in the population, for example, between people living in urban and rural regions [19, 20]. Since attendance rates in the largest cities of the Netherlands are especially low, we decided to focus on urbanized regions. The aim of this study, therefore, was to explore the perspectives concerning cancer screening uptake among inhabitants of The Hague, a highly urbanized region in the Netherlands. Insight in the mechanisms underlying these perspectives could probably be leveraged or applied to promote participation in non-attenders in high urbanized regions.

## Methods

This study was conducted using Q-methodology, a mixed-methods approach designed to provide insight in perspectives on a specific topic in a given population [21, 22]. Q-methodology can be used for a wide range of subjects, and always has to do with the systematic study of subjectivity [23–26]. We conducted the study online due to restrictions following the Covid-19 pandemic.

In brief, respondents were presented with a set of opinion statements on beliefs and motivations for participating in a CSP, and were instructed to rank them according to agreement. Qualitative data was gathered by asking respondents to explain their ranking of the statements and by follow-up interviews with several selected respondents. By-person factor analysis was used to identify significant clusters of correlations among the rankings of statements by respondents. The assumption underlying this analysis is that respondents with similar perspectives on participating in CSPs will rank the statements similarly. For each identified factor, a weighted average ranking of the statements was computed, which was the basis for interpretation and description of the factor as a perspective on cancer screening participation. Selected respondents for each of the factors were invited for a follow-up interview to validate the interpretation of the factors and to obtain additional qualitative data for describing the perspectives [21, 22].

### Statement set development

**Fig. 1:**
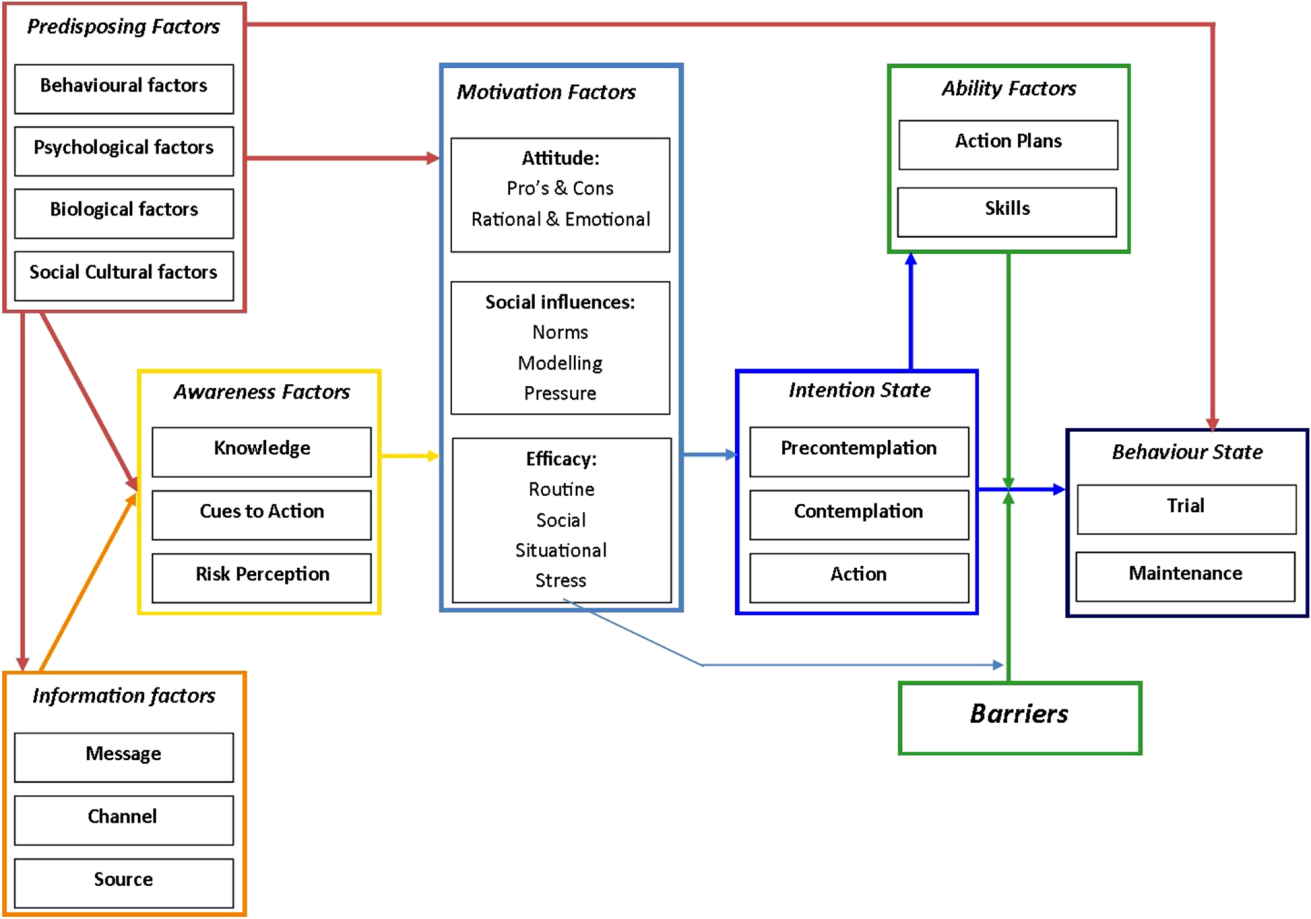
The Integrated Model for Behavioural Change (I-Change Model). The arrows represent the influence between the different factors (referred to as ‘elements’ in the manuscript)

To develop a comprehensive set of statements, representing all the aspects that may be relevant for respondents to express their perspective on the topic, the first two authors (TB, FB) reviewed a large variety of scientific, empirical, and popular literature on motives and beliefs potentially influencing the decision to participate in population-based CSPs. The scientific literature was reviewed systematically and published previously [16]. To structure the statements, and to make sure the set of statements would be comprehensive, the Integrated Change model (I-Change model, Fig. 1) was used as theoretical framework for structuring the development of the statement set [27]. The I-Change model is a health behaviour model, constructed out of several earlier well recognized health behaviour theories, such as: the Health Belief Model, Protection Motivation Theory, Theory of Planned Behaviour and Precaution Adoption Process [28–31]. The I-Change model states that health behaviour is determined by underlying motivations and intentions, and was previously used to study different kinds of health behaviours [32–35]. Since screening attendance can be seen as a (preventive) health behaviour, the elements of the I-Change model provide a useful structure for identifying the aspects that may be relevant for decisions whether or not to participate in a CSP: information, awareness, motivation, ability, intention and barriers. Since predisposing factors (elements) of the I-Change model are more distal factors, more indirectly associated with screening participation, we thought them to be less relevant for including in a Q-study.

Four researchers (TB, FB, MC and VN) developed an initial set of 45 statements based on the collected scientific, empirical, and popular literature. Two external experts were asked to evaluate whether the statement set covered all relevant aspects for the decision to participate in population-based CSPs. Based on their feedback, several adjustments were made; some statements were merged or deleted because they covered similar topics (*n* = 9), some were considered as irrelevant and thus deleted (*n* = 3), and the wording of several statements was revised. Thereafter, we consulted the knowledge institute Pharos (the Centre of Expertise on Health Disparities) [36] to make sure the statements were clear and easily readable for the target population, leading to further reduction of the number of statements (*n* = 2) and minor adjustments to language use. This iterative process resulted in a set of 31 statements. To test the comprehensiveness and clarity of the statement set, a pilot study was conducted among two potential study respondents. Based on their feedback, we finalized the set of opinion statements for the main study.

### Data collection

Due to the outbreak of the Covid-19 pandemic we were not able to perform a face-to-face Q-study, as was the initial plan, and therefore we switched to an online data collection approach. We made use of an external research agency (Flycatcher Internet Research) [37] to recruit respondents. The online data collection was effectuated by making use of the Q Method Software tool [38].

Inhabitants of the city of The Hague, the third largest city of the Netherlands, who were invited for participating in one of the CSPs at least one time, were the target population of this study. The research agency purposively sampled people based on zip-code, sex and age. In total of 112 Inhabitants of the city of The Hague were invited to participate in this study. We focused on the city of The Hague since we were interested in the perspectives of potential cancer screening respondents living in a highly urbanized region, where uptake rates are generally low. Latest attendance rates (2019) of The Hague were 52%, 64%, 57% for the CSPs at cervical, breast and colorectal cancer, respectively [39]. With respect to the demographic characteristics The Hague is comparable to other large cities in the Netherlands, as for example Amsterdam and Rotterdam [40–42].

The invitation to potential respondents included some background information about the study and a link to the online software tool. After following the link, respondents reached a website with detailed instructions and information on the study and data use, including regulations regarding anonymity. By clicking on an ‘agree and start’ button, respondents confirmed to have read and understood the information provided and to take part in the study. Respondents were able to stop participation at any time. In this case, their data was not saved and hence, not included in the study. As it was not possible for respondents to ask for explanation on the ranking process, we provide respondents with extensive clarification materials, both in writing and video before ranking the opinion statements.

**Fig. 2:**
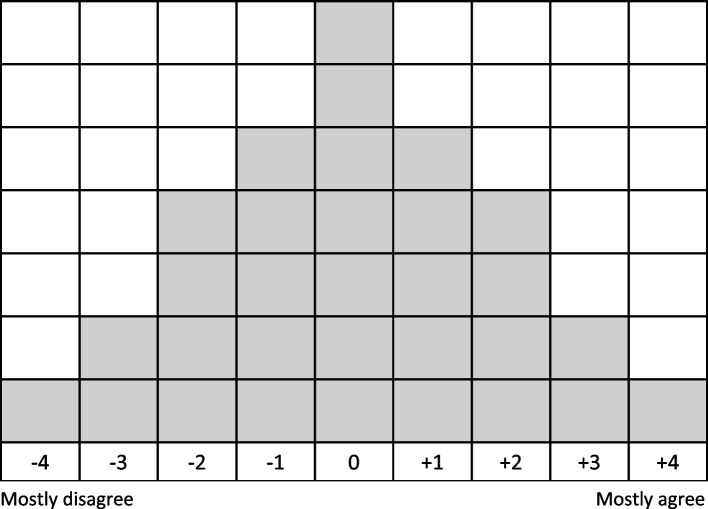
Q-sort grid (9-colum forced choice ranking grid)

During the data collection process, respondents were informed about the study purpose, namely: “We are interested in what you find important when deciding whether or not to participate in a cancer screening programme”. Then, they were presented with the set of opinion statements on participating in the CSPs in random order. First, they were asked to read all the statements and to divide them into three piles (i.e., ‘agree’, ‘neutral’ and ‘disagree’) according to the instruction: “To what extent do you agree with the following statements?”. Next, they were asked to read them again and place them on a forced-choice sorting grid ranging from ‘disagree most’ to ‘agree most’ (see Fig. 2), starting with the statements in the ‘agree’ pile, followed by those in the ‘disagree’ pile and, finally, those in the ‘neutral’ pile. Finally, respondents were asked to review the full ranking of the statements and make any last changes, if desired. Then, they were asked about their demographic details (see Table 1). Finally, respondents were asked to explain their ranking of the statements; in particular, they were asked to explain why they placed the specific statements on both end sides of the ranking grid (i.e., columns -4, -3 and + 3, + 4). After the analysis and initial interpretation of the results, the first author contacted the respondents with the highest factor loadings (i.e., correlation between the ranking of statements by the respondent and the factors) for each factor, to verify the initial interpretation of the factor they were associated with, and to obtain additional qualitative material for finalizing the interpretation and description of the factors. The aim was to interview at least two respondents per factor, so six in total. Respondents then had to leave their contact details in the post-ranking questions. The interviews were audio-recorded after the respondents gave their consent. No data directly leading toward the individual respondent was stored in the audio-file. The interviewed respondents received a €20 gift card for their time investment.

**Table 1:** Characteristics of respondents (*n* = 39)

**Characteristics**		**n**	**%**
Age	30–39	10	25.6
	40–49	3	7.7
	50–59	13	33.3
	60–69	6	15.4
	≥ 70	4	10.3
	Unknown	3	7.7
Sex	Female	28	71.8
	Male	8	20.5
	Unknown	3	7.7
Household	Alone	9	23.1
	Together (partner/children/roommates)	26	66.7
	Unknown	4	10.3
Children	Yes	25	64.1
	No	9	23.1
	Unknown	5	12.8
Education (highest)	Secondary school	5	12.8
	Secondary vocational education	7	17.9
	University of applied sciences	11	28.2
	University	13	33.4
	Unknown	3	7.7
Religion	No	24	61.5
	Christian	10	25.6
	Other religion	1	2.6
	Rather not tell	1	2.6
	Unknown	3	7.7
CSP participation	Yes	31	79.5
	No	5	12.8
	Unknown	3	7.7

*CSP* Cancer screening programme

### Analysis

The data was analysed using KADE version 1.2.0 for MacOS. We excluded respondents of whom the rankings and post-ranking survey answers were in retrospect inconsistent or unclear. This also appeared to be the respondents who completed the ranking exercise very fast, all with a completion time ≤ 8 min (*n* = 6). Furthermore, several responses were excluded based on the answers provided in the post-ranking questions, for example, respondents who indicated that they struggled with the software and had not been able to rank the statements according to instructions. The included respondents completed the raking process with an average time of 25 min, with a maximum of 110 min. In the analysis, first, a correlation matrix of all pairwise correlations between the rankings of the statements by respondents was computed, which was then subjected to by-person factor analysis to identify groups of respondents with mutually high correlations (using centroid factor extraction, followed by varimax rotation). The resulting factors were interpreted and described as perspectives on cancer screening participation. For each factor, a weighted average ranking of the statements was computed (i.e., the factor array), based on the rankings of the statements by the respondents associated with the factor and their factor loadings. In addition, consensus statements (i.e., those whose rankings did not differ significantly between any pair of factors) and distinguishing statements for each factor (i.e., those whose rankings in one factor differed significantly from those in all other factors) were identified. Where consensus statements are suitable for addressing the amount of agreement of the perspectives, the distinguishing statements are useful for highlighting the differences between the different perspectives. Next, an initial interpretation and description of each perspective was based on the factor arrays and the distinguishing and the consensus statements, supplemented with the qualitative data from respondents whose rankings were associated with that perspective (*p* < 0.05).

## Results

**Fig. 3:**
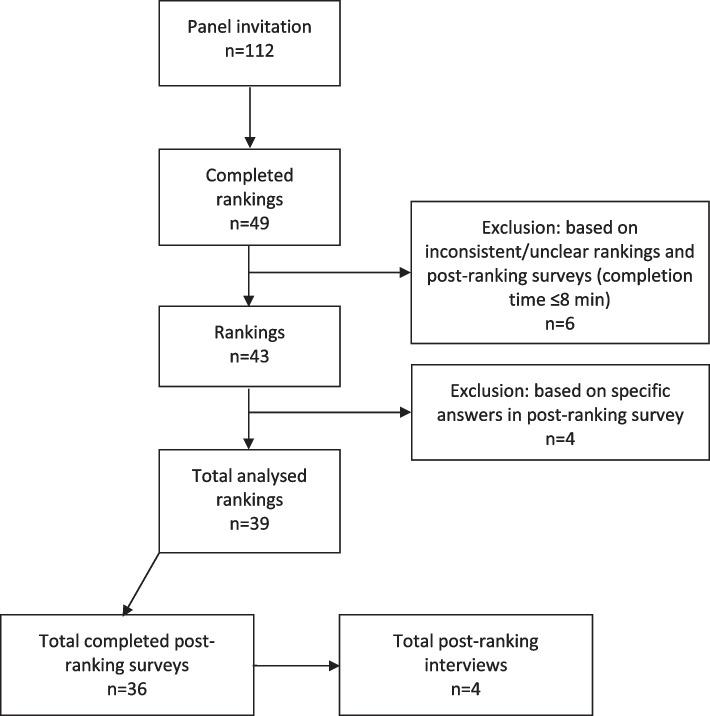
Flowchart on included respondents, rankings of the statement set and qualitative data

Forty-nine respondents (44%) completed the online Q-study, of which 39 rankings (80% of the respondents) were suitable for analysis. Respondents were mostly female and aged between 50 and 59 years of age. CSP participation was defined as participating at least once in a CSP (i.e. respondents who had experience with attending a CSP). Table 1 shows the demographic characteristics of respondents. Thirty-six respondents (92%) completed all the post-ranking questions, so we had missing supplementary data for three of the 39 analysed rankings. The flowchart of the study population is presented in Fig. 3. Afterwards, four post-ranking interviews were conducted. For one factor (perspective 2) none of the respondents left their contact details, so we were not able to perform post-ranking interviews for this perspective. The four interviews lasted about 45 min.

Three distinct perspectives on cancer screening participation were identified based on the ranking data collected. These perspectives were sufficiently distinct and clearly interpretable, based on the qualitative data. Together these perspectives explained 54% of the variance in the ranking of statements by the study respondents, 24%, 10% and 20% for factors 1 to 3, respectively. In total, 32 respondents were significantly associated with one of the factors (*p* < 0.05). Table 2 shows the factor array for each perspective.

**Table 2:** Factor arrays; rank scores per statement for each factor

I-Change elements	Statements	Perspective		
		I	II	III
Information				
1	The invitation for the CSPs is clear to me	+ 2*	+ 1	+ 2
2	I understand the information in the flyer ^a^	+ 1	0	+ 1
3	The flyer helps me deciding on participating in the CSPs	+ 1*	+ 2*	0**
4	The flyer contains information about the advantages AND disadvantages of the CSPs ^a^	+ 1	+ 1	0
5	I have sufficient information about the CSPs to make a choice about attendance	+ 1	+ 3**	+ 1
6	Whenever I have questions about the CSPs I consult my GP	0	+ 3**	0
7	I want my GP to invite me for participating in the CSPs	0	0	-1**
8	I want my GP to provide me with the outcomes of the screening tests	0**	+ 2**	0**
9	I want to receive the screening outcome via post mail ^a^	0	0	+ 1
10	I talk about the CSPs with my partner, children, family, and friends ^a^	+ 1	+ 1	0
11	I would attend an information meeting on the CSPs	0	-1	-2**
Awareness				
12	As long as a do not have any complaints, I do not want to know whether I have cancer	-3	+ 1**	-2
13	There are also disadvantages on participating in a CSP	-1	+ 2**	-1
14	I do believe to have a high risk on developing cancer ^a^	0	0	0
15	By participating in a CSP I can lower my chance of dying as a consequence of cancer	+ 1	0**	+ 2
Motivation				
16	I am afraid to develop cancer	-1**	-2**	+ 3**
17	I think it is important to have a medical check-up now and then, even when I do not have any complaints	+ 4**	-1**	+ 2**
18	I think it is positive that the CSPs are in place	+ 2**	+ 4	+ 4
19	The opinion of my partner, children, family, and friends on participating in a CSP is important to me	+ 1**	-1	-1
20	My faith influences my choice to participate in a CSP ^a^	-2	-3	-3
21	Participating in a CSP does NOT match with my faith ^a^	-3	-3	-4
22	Within my family we do not talk about cancer ^a^	-2	-1	-2
23	By participating in a CSP I am able to do something positive for my health	+ 3	+ 1*	+ 2
Intention				
24	I attend the CSPs because I get invited	+ 2	0**	+ 1
Ability				
25	I think about possible follow-up studies when deciding to participate in a CSP	-1**	0**	+ 1**
Barriers				
26	Participating in a CSP takes a lot of time ^a^	-2	-1	-1
27	I do not participate in a CSP because the follow-up studies cost money	-4**	-2*	-1
28	I have faith in the tests used by the CSPs	+ 3	+ 2*	+ 3
29	None of my peers actually does participate in a CSP ^a^	-2	-2	-3
30	Due to health problems, I am not able to participate in the CSPs	-1*	-4**	-2*
31	The examinations used in the CPS give me an unpleasant feeling	-1	-2	0**

*CSP* Cancer screening programme, *GP* General practitioner

^a^Consensus statement

^*^p < .05, **p < 0.1 versus all other factors

### Perspective 1

Respondents with this perspective hold a positive attitude towards screening. Having regular medical check-ups, even when feeling well, is considered important (statement 17, rank score + 4) and screening attendance is seen as doing sometime positive for your personal health (23, + 3). These respondents think it is important CSPs are in place (18, + 2) and participate because they are invited (24, + 2), the information provided is clear and useful (1, + 2; 2, + 1; 3, + 1; 4, + 1; 5, + 1), and they trust the testing procedure (28, + 3). They also see few disadvantages of participating. The time involved is not a problem for them (26,-2), they are not concerned about potential follow-up testing (25,-1) and any associated costs (27,-4), and they perceive no health (30,-1), or religious objections (21,-3; 20,-2) to participation. Moreover, they do not seem particularly afraid of developing cancer (16,-1; 12,-3) and it is not a taboo topic of conversation in their family (22,-2). In the post-ranking surveys and the interviews, respondents also mainly named advantages of screening attendance. For example, one respondent (ID Z2UT) mentioned: *“Early detection of a possible tumour would lead to earlier treatment, and therefore to better options for cure”*. When potential disadvantages of screening were discussed in the interviews, these were stated as not being relevant enough (ID 2F17): *“Once deviant cells were detected, and as a consequence I had to consult a gynaecologist. Of course, this was not pleasant and I experienced a lot of stress, but the relief afterwards, that it turned out to be good, so I did not have cervical cancer, was much more important. Even though I had a few nights of bad sleep, I would definitely always want to know whether I might have cancer.”* More than in the other two perspectives these respondents tend to value the opinion of people in their social environment about cancer screening (19, + 1), and attending the CSPs was declared to be the social norm (29,-2). “*Among my peers everyone participates with the CSPs. Both my parents and closest friends, all do participate in the CSPs. I actually do not know people who have ethical reasons not to participate”* (ID Z2UT).

We labelled this perspective “positive about participation”. Ten respondents were statistically significantly associated with this perspective, of whom eight reported they participated in CSPs, one reported not participating, and one did not report participation status.

### Perspective 2

Respondents with this perspective are more thoughtful about screening participation. Although these respondents also think it is good that CSPs are in place (18, + 4) and that they can do something positive for their health by participating (23, + 1), they feel there also are disadvantages to participating in screening (13, + 2). Contrary to the other perspectives, these respondents prefer not knowing whether they have cancer as long as they do not have any complaints (12, + 1; 17,-1), and they also have the lowest expectations that participating in screening will lower their risk of dying of cancer (15,0). At the same time, they are least of all afraid of developing cancer (16,-2), compared to the other two perspectives. As one of the respondents explained (ID 1ZCW): *“Without any physical complaints, I do not want to know if a have cancer”.* In addition, several respondents mentioned the possibility of a false-positive and/or false-negative test outcome in the answers of the post-ranking questions. These respondents feel they have sufficient information to make a choice on screening participation (5, + 3; 3, + 2), they trust the testing procedures (28, + 2) and do not perceive health (30,-4), religious (20,-3; 21,-3), or other (27,-2; 29,-2; 31,-2; 26,-1) barriers to participation. Distinctive for this perspective is the role these respondents see for their general practitioner (GP) in cancer screening. In case they would have questions about a CSP, they would first of all consult their GP (6, + 3) and they also would prefer receiving the outcome of a screening test via the GP (8, + 2). One respondent (ID QOIZ) wrote: *“The GP is someone I trust and who is able to provide decent advice on medical issues”.*

We labelled this perspective “thoughtful about participation”. A total of six respondents were statistically associated with this perspective, of whom five reported they participated in CSPs and one reported not participating.

### Perspective 3

Respondents with this perspective think it is good that CSPs are in place (18, + 4), that having regular medical check-ups is important, even when feeling well (17, + 2), and that they can do something positive for their health by participating in CSPs (23, + 2). However, contrary to the other perspectives, these respondents are afraid of developing cancer (16, + 3) and dying as a consequence. They disagree with the statements about not wanting to know whether you have cancer as long as you do not have complaints (12,-2) and that there are also disadvantages to participating in CSPs (13,-1). Most of all respondents they consider follow-up testing in their decision (25, + 1), and reducing the risk of death an important motivation to participate (15, + 2). As one respondent explains (ID IJFC): *“My core motivation for participating in the CSPs is to reduce my chance of dying as a consequence of cancer. I am quite fearful that sooner or later I will get a cancer diagnose. Just the idea of having cancer terrifies me”*. The reason underlying their motivation, also gives them an unpleasant feeling about participation (31,0) (ID IJFC): *“I always find it quite tensive to participate in a CSP. Every time again, I am afraid that they will find something. (…) On the other hand, the fear of a cancer diagnosis out of the blue is even more frightening to me. Therefore, I do participate in the screening programmes”*. These respondents trust the testing procedures (28, + 3), and consider the invitation clear (1, + 2) and a reason to participate (24, + 1). They think the information flyer about screening is not particularly helpful (2, + 1; 3,0; 4;0), however, they would probably not attend a meeting to obtain more information about CSPs (11,-2) (ID 50LC): *“I would never go to an information meeting, or something similar (…) Besides, I do not want to talk with strangers on such delicate topics”*. They feel sufficiently informed to decide about participation (5, + 1) and at any stage do not see a role for their GP (7,-1; 6,0; 8,0) (ID 50LC): *“I do not need any contact with my GP about the CSPs. When I have questions, I will look them up myself. And whenever I need more information, or when something bad has been identified, I do want to discuss this with a specialist in the hospital (…) The GP’s opinion has no added value in this case”.*

We label this perspective “fear drives participation”. A total of 16 respondents were statistically associated with this factor, of whom 12 reported to participate in CSPs, three reported not participating, and one did not report participation status.

### Consensus statements

Several statements were identified as consensus statements (see Table 2), but most of them with scores between + 1 and -1, indicating they were not characteristic for the perspectives (or lack of consensus about them within perspectives). Statements 20 and 21 about religion/faith were generally not seen as barriers to screening participation, nor was statement 26 about partaking in CSPs to be time consuming. Moreover, all perspectives disagreed with statement 29 that most peers do not participate in CSPs.

## Discussion

The aim of this study was to explore the perspectives concerning cancer screening uptake among inhabitants of highly urbanized regions, where participation rates are particularly low. While earlier studies described general characteristics of (non-)attenders, insight in the underlying beliefs and motivations of potential participants regarding cancer screening participation remained limited [16–18]. This study is the first to investigate these underlying beliefs and motivations with respect to cancer screening participation for all three Dutch CSPs together. This provide us insights into the perspectives towards participation in screening in general. Three perspectives were identified using Q-methodology: *“positive about participation”*, “*thoughtful about participation”* and *“fear drives participation”*. The first and third perspective partly overlap in their inclination to participate in CSPs, but significantly differ in the underlying motivation for participating in the CSPs. The second and third perspectives were most distinct from each other.

Both the respondents of the first perspective *(positive about participation*) and third perspective *(fear drives participation)* are likely to participate in CSPs. In the first perspective the motivation and awareness elements of the I-Change model were found to be central. A positive attitude does seem to be linked directly to screening attendance. In literature, attitude is described to be strongly related with intention, and intention, to be medium-strongly related with screening attendance [43]. An overall positive attitude towards the CSPs has been identified as the default among screening eligible people [19, 44, 45]. Together with this positive attitude, respondents of the first perspective participated since it is the social norm, and thereby (probably) also their personal norm. It is known that screening eligible people often feel a kind of moral obligation to attend, and such feelings are recognized as significant predicators for screening attendance [19, 46]. Remarkable was that interviewees with this perspective were not always able to provide correct information on the CSPs and the potential medical follow-up testing. We therefore questioned whether their decision to partake in the CSPs was (always) the result of a well-informed choice, as has been earlier studied by Douma et al., in relation to the publics’ opinion on attending in the colorectal CSP. [47] Thereby, is it known that the benefits regarding CSP participation are most often overestimated (and presented) [48, 49]. In the third perspective motivation elements of the I-Change model were the most important. Respondents attended the CSPs based on feelings of fear and unpleasantness. Such negative emotions were earlier already described as to both facilitate as deter cancer screening attendance [50–52]. In an earlier study we identified feelings of inconvenience, insecurity and anxiety towards the screening tests and outcomes, as determinants of low or non-attendance [16]. In this study, respondents with the third perspective revealed that an underlying fear, such as worrying to die from cancer, could also be a motivator for screening attendance. Exclusive for this perspective are the comments of the respondents on all knowing people who actually suffered or died as a consequence of cancer. This implies respondents experienced the effects of a cancer diagnosis directly, and therefore feel more susceptible to be diagnosed with cancer. This is most probably also influencing the risk perception of these people. Several health behaviour modules, including the I-Change model, postulate that risk perception motivates screening attendance. In literature there is no consensus regarding this topic, however most recent studies report on, a small positive association of risk perception and screening attendance [53–55]. A last distinctive component of the third perspective is their tendency to be less open for external influence and guidance. This could be an important issue when trying to reach out to people holding this perspective, for example by healthcare professionals or policy makers.

People within the second perspective (*thoughtful about participation*) appeared to be more hesitant in making a decision about participating in cancer screening. Therefore, they can be considered critical regarding CSP participation. Key in this perspective are the awareness and information elements of the I-Change model. In contrast to the other two perspectives respondents doubted the effectivity of CSPs, and think potential consequences of screening (inter alia false-positive and false-negative test outcomes) participation are more important. These finding relate to the protection motivation theory of Rogers, in which response efficacy and response cost are acknowledged as having an effect on screening attendance [29]. Answers in the post-ranking questions suggested respondents were better informed on the possible consequences of the CSPs. This perspective might be related to a need for autonomy as described in a recent study [56]. However, our qualitative data, in particular, revealed that participants think about the potential disadvantages of participating and know that screening is not always conclusive. For this reason, we think our participants are more “thoughtful about participation” than that they have a need for autonomy. Unique in this perspective is the role respondents see for their GP as advisor. Previous studies showed that involvement of primary care leads to an increase of screening attendance rates [57, 58], in particular among lower socioeconomic and minority groups [59, 60]. This primary care involvement could therefore also be preferred by people who are (more) thoughtful on participation, and thus might be independent of the socioeconomic position in society.

Due to several (practical) choices this study has some limitations. First, a Q-methodology study has an exploratory nature and can be used to identify and describe the main perspectives on a topic in a certain population. The sampling strategy used in Q-methodology studies, is however not informative about how common these perspectives are among people eligible for cancer screening participation in general (frequency question), nor how the perspectives are associated with the characteristics of respondents, or why specific respondents with the same perspective present different screening behaviour [61]. Such ‘frequency-questions’ could be examined with surveys, [62] whereas future ‘how and why-questions’ can be answered by performing additional interviews and focus groups [63]. Second, respondents were recruited from an existing research panel of an external agency. On the one hand this allowed us to conduct the study remotely and thereby guaranteeing full anonymity, whereby respondents did not feel any social pressure during the ranking exercise. On the other hand, it introduced a selection and led to several specific drawbacks. Our sample predominantly contained women, aged between 50 and 69 years, living with a partner, and were higher educated (Table 1). From literature it is known that people with these characteristics are more prone to participate in the CSPs [16]. When taking the general demographics of the screening eligible inhabitants of The Hague into account, one would expect to included: more men, more people living alone, lesser people with children, more people with vocational education or lower, and more people who adhere to a religion [40]. It is possible that additional perspectives would have been identified if more respondents with these more general characteristics had been included in this study. Therefore we recommend future studies with a similar aim to use a face-to-face sampling approach. Furthermore, the switch to the online data approach may have affected the number of exclusions as issues with the software tool that were not addressed in the explanation materials could not been solved. And, lastly, it was not possible to obtain an interview with the two respondents most strongly associated with each factor directly after they had finished their ranking of the statements, as they could only be invited for this interview after all data was collected and the analysis was finalized. Third, statement categorization by the I-Change model was challenging, especially since the relationship between the components is not always clearly defined [27, 32]. Respondents are not familiar with the subdivision of the I-Change model and could therefore classified some statements differently. However, since we upfront tested our statement set and none of the initial potential respondents, nor the actual respondents, reported to mis significant statements important to their perspective, we believe the I-Change model to be suitable in order to create a comprehensive set of statements.

This Q-methodology study shows that beliefs and motivations towards CSPs are not only different between attenders and non-attenders, but can also differ between subgroups of people holding different perspectives. In order to increase awareness and knowledge regarding the CSPs, we therefore suggest tailoring communications to the perspectives of potential participants. This implies that for perspective 1 more attention needs to be paid to providing informing about the CSPs and follow-up medical testing procedures, that for perspective 2 more attention needs to pe paid to the potential disadvantages of screening, and that for perspective 3 to more education needs to be provided about risks and numbers relating morbidity and mortality. For two of the perspectives in this study, communication channels others than the GP were found to be appropriate. However, for the respondents of the second perspective, who doubted screening attendance and thought about the potential consequences of the screening, information provided by a GP, or a perhaps another trusted primary care health professional, seems essential.

## Conclusions

Conducting this study allowed us to explore the perspectives of people living in a highly urbanized region concerning cancer screening participation. Our study identified three perspectives on beliefs and motivations underlying screening attendance. Since CSPs will only be effective when participation rates are sufficiently high, it is essential to have insight into the different perspectives among potential respondents concerning partaking in a CSP. Tailor-made communication strategies for these different perspectives are highly recommended to increase awareness and knowledge regarding the CSPs, and probably should also involve primary care health professionals, at least for a part the population. The findings of this study could contribute to the future optimization of the CSPs.

## Data Availability

The datasets used and/or analysed during the current study is available from the corresponding author on reasonable request.

## Abbreviations

CSP: Cancer Screening Programme
RIVM: National Institute for Public Health and the Environment
WHO: World Health Organization
I-Change model: Integrated Change model
GP: General Practitioner
