# Publisher Correction: Perspectives on cancer screening participation in a highly urbanized region: a Q-methodology study in the Hague, the Netherlands

**DOI:** 10.1186/s12889-022-14499-6

**Published:** 2022-11-04

**Authors:** Thomas H.G Bongaerts, Frederike L. Büchner, Matty R. Crone, Job van Exel, Onno R. Guicherit, Mattijs E. Numans, Vera Nierkens

**Affiliations:** 1 Health Campus The Hague, Leiden University Medical Center, The Hague, The Netherlands; 2 Department of Public Health and Primary Care, Leiden University Medical Center, Leiden, The Netherlands; 3 Erasmus School of Health Policy & Management, Erasmus University Rotterdam, Rotterdam, The Netherlands; 4 Erasmus Centre for Health Economics Research, Erasmus University Rotterdam, Rotterdam, The Netherlands; 5 Haaglanden Medical Center, University Cancer Center Leiden, The Hague, The Netherlands

**Correction: BMC Public Health (2022) 22:1925**

10.1186/s12889-022-14312-4

During the publication process a typo was introduced in this article. The correct and incorrect information is shown below. The original article has been updated. The publisher apologizes for the inconvenience caused to the authors & readers.

**Incorrect**

For this reason, we think our participants are more "positive about participation", "thoughtful about participation" than that they have a need for autonomy.

**Correct**

For this reason, we think our participants are more "thoughtful about participation" than that they have a need for autonomy.

